# Head-to-head preclinical treatment design prioritizes promising therapies for neurofibromatosis type 1 optic glioma clinical translation

**DOI:** 10.1093/noajnl/vdaf215

**Published:** 2025-10-04

**Authors:** Talia Eligator, Jit Chatterjee, Shintaro Yamada, Anthony Kirchner, Hareesh B Nair, Jason R Fangusaro, David H Gutmann

**Affiliations:** Department of Neurology, Washington University School of Medicine, Saint Louis, MO (T.E., J.C., S.Y., A.K., D.H.G.); Department of Neurology, Washington University School of Medicine, Saint Louis, MO (T.E., J.C., S.Y., A.K., D.H.G.); Department of Neurology, Washington University School of Medicine, Saint Louis, MO (T.E., J.C., S.Y., A.K., D.H.G.); Department of Neurology, Washington University School of Medicine, Saint Louis, MO (T.E., J.C., S.Y., A.K., D.H.G.); Department of Molecular and Translational Medicine, Texas Tech University Health Science Center, Rick Francis St, El Paso, Texas, 79905 (H.B.N.); Department of Pediatrics, Children’s Healthcare of Atlanta and the Aflac Cancer Center, Emory University School of Medicine, Atlanta, Georgia; Department of Neurology, Washington University School of Medicine, Saint Louis, MO (T.E., J.C., S.Y., A.K., D.H.G.)

**Keywords:** carboplatin, lamotrigine, low-grade glioma, neurofibromatosis, optic glioma, pediatric brain tumor, vision

## Abstract

**Background:**

Authenticated preclinical brain tumor models provide unprecedented opportunities to evaluate next-generation treatments. However, some therapies with robust anti-tumor activity in mice fail in human trials, highlighting the need to better prioritize candidates for clinical translation. Herein, we implemented a head-to-head preclinical strategy using a well-characterized murine model of NF1-optic pathway glioma (*Nf1*^OPG^).

**Methods:**

*Nf1* ^OPG^ mice were treated with standard of care (SOC; carboplatin), clinically evaluated (everolimus, mirdametinib), and investigational (pexidartinib, HBS-101, lamotrigine) drugs during the period of most rapid tumor growth (6-12 weeks of age). Anti-tumoral efficacy was assessed by proliferation (%Ki67^+^ cells) and optic nerve (ON) volume, while vision-related outcomes were measured using retinal nerve fiber layer (RNFL) thickness and retinal ganglion cell (RGC) determinations. Tumor microenvironment (TME) soluble mediator (Ccl2, Ccl3, Ccl4, Ccl5) and tumor cell marker (NeuN, Gpr17) RNA expression was quantitated by qRT-PCR. Outcomes were compared to carboplatin-treated *Nf1*^OPG^, untreated *Nf1*^OPG^, and *Nf1^+/-^* mice.

**Results:**

While all agents restored normal tissue architecture, reduced ON proliferation, and decreased TME soluble mediator and tumor cell marker RNA expression, only lamotrigine and mirdametinib also reduced ON volume. Everolimus, lamotrigine, and HBS-101 restored RNFL thickness to wild-type levels, whereas carboplatin showed a trend towards normalization.

**Conclusions:**

This referential preclinical study design affords direct head-to-head comparisons of investigational therapies relative to SOC treatment using clinically meaningful outcomes (OPG growth and RNFL thickness). Using this strategy, lamotrigine emerged as the most promising therapy for limiting tumor progression and vision loss in *Nf1*-OPG mice, relevant to clinical translation for children with NF1-OPG.

Key Points

- Standard and molecularly targeted therapies all similarly reduced tumor proliferation.
- Everolimus and MDK inhibitor treatments significantly decreased RNFL thinning.
- Only lamotrigine reduced OPG volume, tumor proliferation, and RNFL thinning.

Importance of the Study

With the development of authenticated preclinical brain tumor models, the ability to identify and prioritize promising drugs for clinical translation has been greatly accelerated. However, many of the drugs demonstrating excellent anti-tumor responses in mice have not demonstrated efficacy in human clinical trials. To develop a referential strategy to nominate high-priority candidates for translation, we leveraged an accurate and authenticated preclinical murine avatar of NF1-associated low-grade glioma (optic pathway glioma; OPG). By comparing anti-tumoral and stromal therapies head-to-head with the one of the most commonly used chemotherapy agents, carboplatin, and assessing the effects on tumor proliferation and volume, as well as a surrogate measure of visual acuity, we demonstrate that lamotrigine, targeting neuron-mediated stromal support, was the most overall efficacious treatment for NF1-OPG. We propose that future drug efficacy evaluations consider direct comparisons to conventional standard-of-care therapy using relevant clinical and functional outcomes for children and adults with brain tumors.

The poor translation of preclinical studies to the clinical workplace is often framed as a failure of mouse tumor modeling to predict therapeutic responses in humans.^1^ In this regard, numerous promising molecularly targeted therapies that exhibited robust anti-tumoral responses in rodents (mice and rats) lacked similar efficacy in clinical trials. While these disappointing results could reflect innate differences between rodents and humans (e.g., pharmacokinetics, brain size) or the use of dissimilar outcome measures (e.g., bioluminescence), they might also result from a lack of comparisons to conventional first-line therapies or the incorporation of meaningful functional outcomes.^2^ Such discrepancies may also reflect statistically, but not clinically, significant shifts in survival curves, as well as differences in treatment dosing used in preclinical versus clinical settings.

To determine whether a head-to-head referential approach might better prioritize therapies for clinical translation,^3^ we leveraged an authenticated and well-characterized small animal model of the most common brain tumor (optic pathway glioma; OPG) encountered in children with the Neurofibromatosis type 1 (NF1) cancer predisposition syndrome.^4^^,^^5^ In the setting of NF1, 15%-20% of young individuals will develop a non-fatal OPG, most frequently involving the anterior optic pathway (optic nerve and chiasm).^6^^,^^7^ These tumors are typically low-grade (World Health Organization grade 1 astrocytomas) neoplasms with low proliferative indices (1-4% Ki67^+^ cells).^5^ Since these tumors arise along the optic pathway, important for transmitting visual information to the brain, nearly one-third of affected children will experience a reduction in visual acuity^8^ associated with thinning of the retinal nerve fiber layer (RNFL), which can be objectively ascertained by optical coherence tomography.^9^ When visual acuity is reduced on sequential ophthalmologic assessments, children with NF1-OPG are typically treated with chemotherapy, and therapeutic success is based on both tumor shrinkage and vision stabilization/improvement.^10^ Since the majority of children will survive their tumor, a main focus in the modern era has been to prioritize functional outcomes, such as vision, motor abilities, neurocognitive outcomes, and quality of life.^11^

First line treatment for NF1-OPG is usually a combination of carboplatin and vincristine, where carboplatin is thought to be the major anti-tumoral compound.^12^ However, a proportion of children are unable to tolerate carboplatin due to allergic reactions or hematologic sequalae^13^ and require an alternative therapeutic regimen. While some centers use vinblastine,^14^ there has been a shift towards the use of molecularly targeted therapies, specifically MEK inhibitors^11^^,^^15^ based on promising efficacy noted in early phase 1 and 2 clinical trials. MEK inhibitors target one of the main mitogenic pathways increased in *NF1*-mutant brain tumors.^5^ However, numerous other agents, targeting additional tumor cell signaling pathways (eg mTOR^16^) or non-neoplastic cell types in the supportive tumor microenvironment (pexidartinib, tumor associated monocytes;^17^ lamotrigine, neurons^18^^,^^19^) have also been explored as potential treatments in preclinical mouse models.

To develop a strategy for prioritizing compounds for future clinical translation, we used *Nf1*^OPG^ mice, which develop low-grade astrocytomas within the prechiasmal optic nerves and chiasm by 3 months of age associated with RNFL thinning and eventual reductions in visual acuity.^4^^,^^5^ We specifically chose to treat these *Nf1*^OPG^ mice with human-comparable treatment dosing between 6 and 12 weeks of age, the period when the greatest increase in glioma growth is observed, indicating that the intervention attenuates tumor growth rather than prevents optic glioma initiation,^20^ and measured tumor proliferation and volume, as well as RNFL thickness as a surrogate for vision loss. Using this preclinical design framework, we compared other investigational therapies to carboplatin and highlighted the advantages and disadvantages of each therapy for their tumor-suppressive and vision restorative properties. Moreover, this head-to-head comparative strategy allowed us to identify lamotrigine as the most efficacious preclinical treatment based on both its potent anti-tumoral and neuroprotective effects.

## Materials and Methods

### Mice

*Nf1* ^flox/mut^; GFAP-Cre (*Nf1*^OPG^) mice, *Nf1*^+^^*/*^^-^ mice, and wild type (WT) mice were used under an approved Animal Studies Committee protocol at Washington University in St Louis. All mice used in these experiments were maintained on a strict C57BL/6 background with controlled light-dark cycles (12h:12 h) with *ad libitum* access to food and water.

### In Vivo Mouse Treatments

*Nf1* ^OPG^ mice were treated from six to twelve weeks of age with carboplatin (15 mg/kg, IP, once weekly, dissolved in saline with 1% methylcellulose, Sigma-Aldrich C2538), everolimus (1.7 mg/kg, oral gavage daily, dissolved in PBS, Selleck Chemicals S1120), mirdametinib (PD0325901; 2.5 mg/kg, oral gavage twice daily for five days per week, reconstituted from 10 mg powder in 500 μL PBS, STEMCELL Technologies 72184), lamotrigine (2.5 mg/kg, oral gavage twice daily for five days per week, dissolved in saline with 1% methylcellulose, Selleck Chemicals S3024), HBS-101 (20 mg/kg, IP, five days per week, dissolved in PBS), or pexidartinib (275 mg/kg, PLX3397-containing chow daily, Medchem HY-16749A). We treated mice between 6 and 12 weeks of age, during the period of most rapid tumor growth; after this period, tumor volume and proliferation stabilizes, indicating that the intervention suppresses tumor growth rather than reverses tumor development.

### Optic Nerve Volume Analysis

Dissected optic nerves were photographed using a Leica S9D Flexcam C3 microscope. Images were analyzed using NIH ImageJ. Measurements at the chiasm 0μm (D_1_), 150μm (D_2_), 300μm (D_3_), and 450μm (D_4_) anterior to the chiasm were used to generate optic nerve volume (V) estimates using the following formula in each of the three segments between the four points of reference (D_1_D_2_; D_2_D_3_; D_3_D_4_): V_1_ = 1/12πh ($D_{1}^{2}$ + D_1_ • D_2_ + $D_{2}^{2}$), where V_1_ = the volume of the first segment, h = 150 μm, and D_1_ = diameter of the first segment. The sum of the three segments (V_1_ + V_2_ + V_3_) was ultimately used to calculate the total optic nerve volume as previously described.^18^

### Immunohistochemistry and Immunofluorescence

Mice were euthanized and perfused with Ringer’s solution followed by 4% paraformaldehyde. Optic nerves were collected and embedded in paraffin, while eyes were harvested and embedded in 2:1 sucrose: OCT. 5-μm-thick optic nerve paraffin sections or 10-μm-thick eye cryosections were immunostained with primary antibodies (Supplementary Table 1). Development was performed using the Vectastain ABC kit (Vector Laboratories, PK4000) and biotinylated secondary antibodies (Vector Laboratories) or Alexa-fluor-conjugated secondary antibodies (Supplementary Table 1). Slides were imaged with a Leica ICC50W microscope using LAS EZ software or a Thunder Imager 3D microscope using LAS AF Lite 3.2.0 software, where quantification was performed as previously reported.^18^

### RNA Extraction and Real-Time RT-qPCR

Mice were euthanized using isoflurane followed by cervical dislocation, and the optic nerves were collected. RNA was isolated from optic nerves using the NucleoSpin RNA Plus kit (Takara-740984.205). Optic nerves from two individual mice were pooled (total = 2 optic nerves and 2 chiasms) to obtain sufficient cDNA for downstream experiments. Total RNA was reverse transcribed into cDNA using the High-Capacity cDNA Reverse Transcription Kit (Applied Biosystems, 4368814). Real-time quantitative PCR (RT-qPCR) was performed by TaqMan gene expression (*Ccl2*, *Ccl3*, *Ccl4*, *Ccl5*, *Gapdh*, *Gpr17*, *Neu4*; Supplementary Table 2) using the TaqMan Fast Advanced Master Mix (Applied Biosystems). All reactions were performed using the QuantStudio 3 (Applied Biosystems) system. ΔΔCT values were calculated using *Gapdh* as an internal control as previously reported.^18^

### Human and Mouse Single Cell Analysis

Raw sequencing data from mouse *Nf1*^OPG^ optic nerve samples were processed using the 10X Genomics Cell Ranger pipeline (version 6.1.1) to generate gene count matrices, which were aligned to the mm10-2020, a mouse reference genome. These data were merged with previously published mouse optic nerve datasets (GSM7816061 and GSM7816062; GSE244433), resulting in a total of four individual mouse *Nf1*^OPG^ tumor samples. For the human pediatric pilocytic astrocytoma (PA) tumors, five separate tumors (GSM7816067-GSM7816071; GSE244433) were included. All datasets were analyzed using the Seurat R package (version 5.2.1). Cells were excluded if they had fewer than 500 cells, more than 5000 detected genes, more than 5%-10% mitochondrial gene content (5% for mouse, 10% for human), or fewer than 500 or more than 20,000 transcript counts. Log normalization was performed using the NormalizeData function, and 2000 highly variable features were identified and scaled using FindVariableFeatures and ScaleData. Principal component analysis (PCA) was performed with RunPCA, followed by data integration with the IntegrateLayers function using CCAIntegration. The integrated data were subjected to dimensional reduction and clustering with RunUMAP, FindNeighbors, and FindClusters, and cell types were assigned based on canonical marker genes.

To identify potential tumor-specific markers, differential gene expression analysis was performed comparing tumor cells and each non-neoplastic cell type using the FindMarkers function in Seurat. For each comparison, genes were selected based on the following criteria: (1) expression in >45% of tumor cells (pct.1 > 0.45), (2) expression in <2% of cells from the other cell type (pct.2 < 0.02), and (3) an average log2 fold change greater than 4.5 (Supplementary Table 3). Genes meeting these thresholds in each pairwise comparison were considered tumor-specific markers and further analyzed by qRT-PCR on independently generated sample.

### Quantification and Statistical Analysis

Data analysis was performed using GraphPad Prism software (version 10). Differences between treated groups and the untreated group were determined using an unpaired *t*-test. Multiple comparisons were analyzed using a one-way analysis of variance (ANOVA). Statistical significance was defined as *P* ≤ 0.05.

## Results

### Carboplatin Treatment Reduces Tumor Proliferation in *Nf1* ^OPG^ Mice

**Figure 1.**
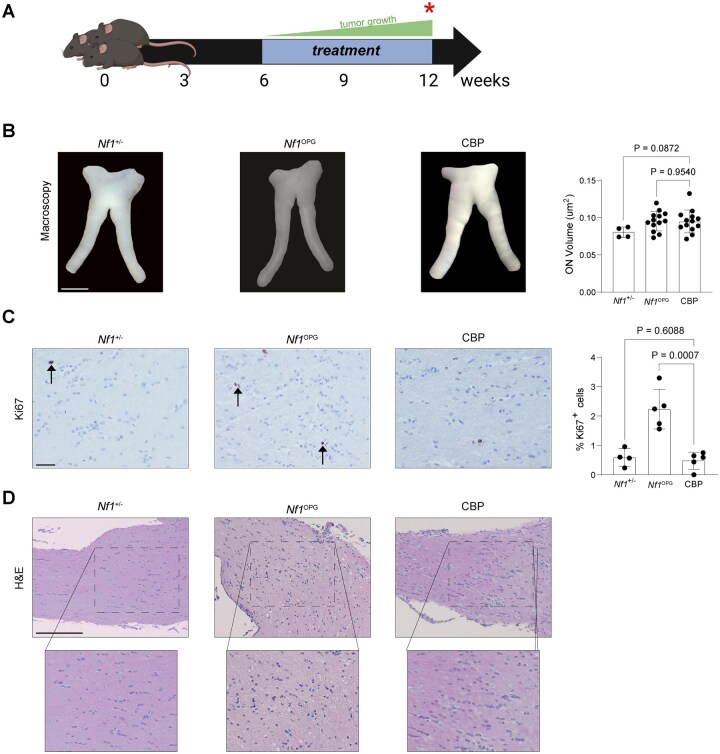
(A) *Nf1*^OPG^ mice were treated with carboplatin (Sigma-Aldrich C2538; 15mg/kg IP injection once weekly) from six to twelve weeks of age. Optic nerves were analyzed following euthanasia at 12 weeks of age (red asterisk). (B) Optic nerve volume analysis (*Nf1^+/-^*, *n* = 4 mice; *Nf1*^OPG^, *n* = 13 mice; CBP, *n* = 13 mice). (C) Immunohistochemistry (IHC) and quantification of tumor cell proliferation (%Ki67^+^ cells in brown; *Nf1^+/-^*, *n* = 4 mice; *Nf1*^OPG^, *n* = 5 mice, CBP, *n* = 5 mice). (D) Optic nerve cellularity and tissue architecture are demonstrated on H&E staining. Scale bars, (B), 1mm; (C), 40µM; (D), 40µm. Data are represented as the mean ± SEM. (B-C), Two-tailed student’s *t*-test between treated and untreated groups. *P*-values are indicated within each graph. Figure illustrations (1A) were created using BioRender (BioRender.com).

To evaluate the efficacy of standard-of-care carboplatin (CBP) monotherapy, *Nf1*^OPG^ mice were treated from 6 to 12 weeks of age, during the period of increasing tumor growth,^20^ and euthanized at 12 weeks of age (Figure 1A). At 12 weeks of age, while CBP-treated *Nf1*^OPG^ mouse nerve volumes were unchanged (0.99-fold change; Figure 1B), there was a reduction in optic nerve tumor proliferation to levels comparable to *Nf1^+/-^* mice, as measured by the percentage of Ki67^+^ cells, compared to untreated *Nf1*^OPG^ mice (Figure 1C). *Nf1^+/-^* mice were used for comparison to represent the genetic composition of children with NF1 (harboring only a germline inactivating *NF1* gene mutation). We specifically chose comparisons to untreated, rather than vehicle-treated, mice to more accurately reflect clinical practice where many children with NF1-associated low-grade glioma are monitored and untreated.

Consistent with reduced tumor proliferation, there was decreased tumor-associated architectural tissue distortion (Figure 1D). In addition, CBP treatment had no effect on apoptosis (%caspase-3^+^ cells; Supplementary Figure 1A) compared to *Nf1*^OPG^ mice and minimal effects on tumor-associated monocytes (TAM; %Iba1^+^ cells; Supplementary Figure 1B), but reduced CD8^+^ T cell (Supplementary Figure 1C), Olig2^+^ cell (Supplementary Figure 1D), and BLBP^+^ cell (Supplementary Figure 1E) content to levels comparable to those seen in *Nf1^+/-^* mice.

### Clinically Evaluated Targeted Therapies Exhibit Similar Efficacy to Carboplatin in *Nf1* ^OPG^ Mice

**Figure 2.**
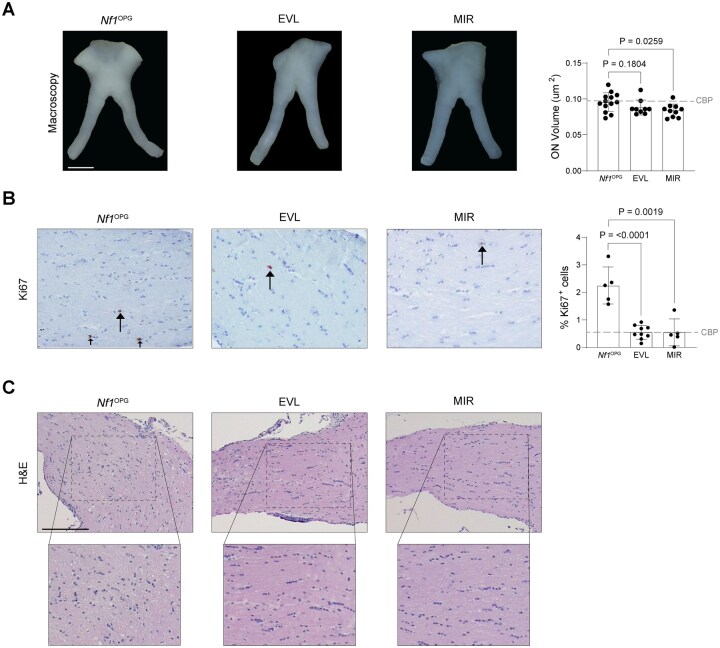
*Nf1* ^OPG^ mice were treated with everolimus (EVL; Selleck Chemicals S1120; 1.7 mg/kg gavage daily) or mirdametinib (MIR; STEMCELL Technologies 72184; 2.5mg/kg oral gavage twice daily for five days each week) from six to twelve weeks of age. Optic nerves were analyzed following euthanasia at 12 weeks of age. (A) Immunohistochemistry (IHC) and quantification of tumor cell proliferation (%Ki67^+^ cells in brown; *Nf1*^OPG^, *n* = 5 mice; EVL, *n* = 9 mice; MIR, *n* = 5 mice). Dotted lines denote the average value from carboplatin (CBP)-treated mice. (B) Optic nerve volume analysis (*Nf1*^OPG^, *n* = 13 mice; EVL, *n* = 9 mice; MIR, *n* = 10 mice). Dotted lines denote the average values from carboplatin (CBP)-treated mice. (C) Optic nerve cellularity and tissue architecture are demonstrated on H&E staining. Scale bars, (A), 1 mm; (B), 40 µM; (C), 40 µM. Data are represented as the mean ± SEM. (B-C) Two-tailed student’s *t*-test between treated and untreated groups. *P*-values are indicated within each graph.

Based on prior preclinical studies targeting RAS pathway signaling molecules (MEK,^5^ mTOR^21^), mirdametinib and everolimus, respectively, have entered clinical trials for children with low-grade glioma, including those with NF1-OPG [NCT01158651; NCT04923126].^16^ To compare mirdametinib and everolimus to standard-of-care therapy (CBP), *Nf1*^OPG^ mice were treated with clinically comparable doses of everolimus (EVL; 1.7 mg/kg gavage daily) or mirdametinib (MIR; 2.5mg/kg oral gavage twice daily for five days each week) from 6 to 12 weeks of age. At 12 weeks of age, mirdametinib, but not everolimus, treatment reduced optic nerve volume relative to untreated mice (EVL, 0.92-fold change; MIR, 0.87-fold change; Figure 2A). In contrast, both treatments reduced proliferation (%Ki67^+^ cells) comparable to CBP-treated mice, relative to untreated controls (Figure 2B). Additionally, both treatments resulted in reduced tumor-related architectural tissue distortion (Figure 2C), CD8^+^ T cell infiltration (Supplementary Figure 2A), Olig2^+^ cell content (Supplementary Figure 2B), and BLBP^+^ cell content (Supplementary Figure 2C). As observed in other preclinical experiments using *Nf1*-OPG mice,^5^^,^^18^^,^^20^ TAM (%Iba1^+^ cells) content was not altered after treatment (Supplementary Figure 2D).

### Investigational Therapies Also Reduce *Nf1*-OPG Growth

We previously demonstrated that murine *Nf1*-OPG growth is tightly regulated by neuron-immune-tumor interactions, such that *Nf1*-mutant neurons, specifically retinal ganglion cells (RGCs), induce T cell-TAM stromal support through the expression of midkine (Mdk), a secreted growth factor.^22^ Since Mdk expression is modulated in a neuronal activity-dependent manner, we showed that lamotrigine (LTR), a hyperpolarization-activated cyclic nucleotide-gated channel (HCN) agonist, inhibits Mdk expression.^18^

**Figure 3.**
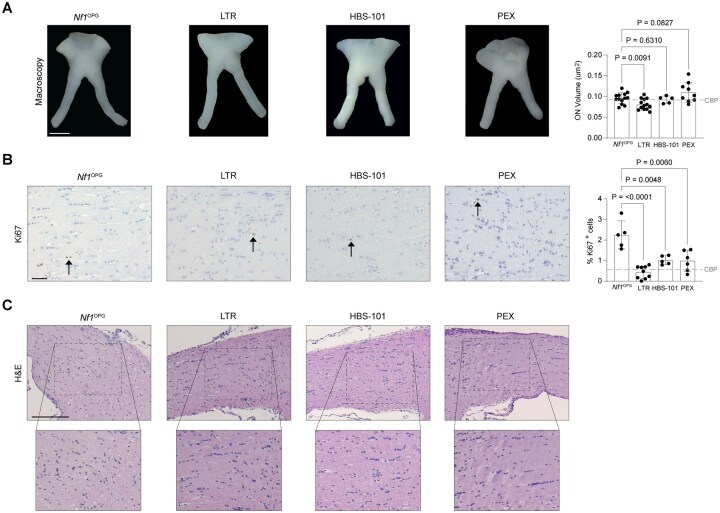
*Nf1* ^OPG^ mice were treated with lamotrigine (LTR; Selleck Chemicals S3024; 2.5mg/kg gavage twice daily for five days each week), HBS-101 (20 mg/kg IP injection for 5 days each week), or pexidartinib (PEX; 275 mg/kg PLX3397-containing chow daily) from six to twelve weeks of age. Optic nerves were analyzed following euthanasia at 12 weeks of age. (A) Immunohistochemistry (IHC) and quantification of tumor cell proliferation (%Ki67^+^ cells in brown; *Nf1*^OPG^, *n* = 5 mice; LTR, *n* = 9 mice; HBS-101, *n* = 5 mice; PEX, *n* = 6 mice). Dotted line denotes average value from carboplatin-treated nerves. (B) Optic nerve volume analysis (*Nf1*^OPG^, n = 13 mice; LTR, *n* = 13 mice; HBS-101, *n* = 5 mice; PEX, *n* = 9 mice). Dotted lines denote the average values from carboplatin (CBP)-treated mice. (C) Optic nerve cellularity and tissue architecture are demonstrated on H&E staining. Scale bars, (A), 1 mm; (B), 40 µM; (C), 40 µM. Data are represented as the mean ± SEM. (B-C), Two-tailed student’s *t*-test between treated and untreated groups. *P*-values are indicated within each graph.

To evaluate the efficacy of TME-targeted therapies, *Nf1*^OPG^ mice were treated from 6 to 12 weeks of age with lamotrigine^18^ (LTR) and HBS-101^23^ to block midkine bioavailability or pexidartinib (PLX3397), a CSF1R inhibitor targeting TAM. At 12 weeks of age, only lamotrigine treatment reduced optic nerve volume (LTR, 0.84-fold change; HBS-101, 0.96-fold change; PEX, 1.15-fold change; Figure 3A). In contrast, optic nerves from all treated mice exhibited reduced proliferation (%Ki67^+^ cells; Figure 3B) and cellular distortion (Figure 3C) relative to untreated controls, which was comparable to that observed following carboplatin treatment. Similarly, lamotrigine, HBS-101, and pexidartinib treatments reduced CD8^+^ T cell infiltration (Supplementary Figure 3A) and BLBP^+^ cell content (Supplementary Figure 3B), whereas HBS-101 and exidartinib, but not lamotrigine, reduced Olig2^+^ cell content (Supplementary Figure 3C). Consistent with its known effects on TAM depletion,^24^^,^^25^ only pexidartinib decreased TAM (%Iba1^+^ cells; Supplementary Figure 3D) content.

### Single-Cell Transcriptomics Identifies Tumor-Specific Markers

**Figure 4.**
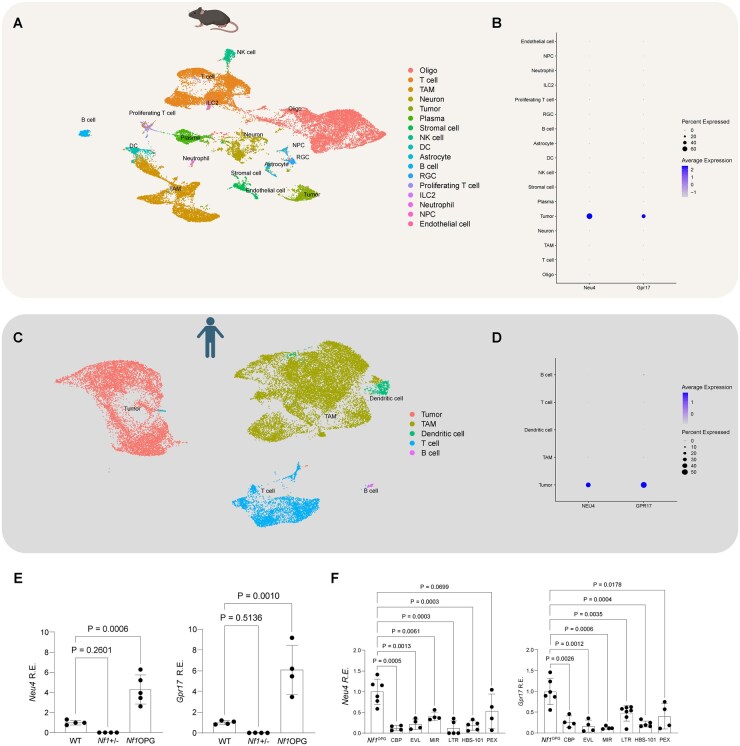
(A) t-SNE visualization plot showing single-cell RNA sequencing results from *Nf1*^OPG^ mice. (B) Mouse dot plot showing shared upregulated genes in the tumor clusters of single-cell RNA sequencing data from *Nf1*^OPG^ mice and human pilocytic astrocytoma (PA) tumors. (C) t-SNE visualization plot showing single-cell RNA sequencing results from human PA tumors. (D) Human dot plot showing shared upregulated genes in the tumor clusters of single-cell RNA sequencing data from *Nf1*^OPG^ mice and human PA tumors. (E) *Neu4* (WT, *n* = 4 mice; *Nf1*^+/-^, *n* = 4 mice, *Nf1*^OPG^, *n* = 5 mice) and *Gpr17* (*n* = 4 mice) mRNA expression by qPCR in *Nf1*^+/-^ and *Nf1*^OPG^ mice relative to wild-type mice. (F) *Neu4* (*Nf1*^OPG^, *n* = 6 mice; CBP, *n *= 4 mice; EVL, *n* = 4 mice; MIR, *n* = 4 mice; LTR, *n* = 7 mice; HBS-101, *n* = 5 mice; PEX, *n* = 4 mice) and *Gpr17* (*Nf1*^OPG^, *n* = 6 mice; CBP, *n* = 4 mice; EVL, *n* = 4 mice; MIR, *n* = 4 mice; LTR, *n* = 5 mice; HBS-101, *n* = 5 mice; PEX, *n* = 4 mice) mRNA expression by qPCR in treated and untreated *Nf1*-OPG mice. Data are represented as the mean ± SEM. (E), One-way ANOVA with Dunnett’s posttest correction. (F), Two-tailed student’s t-test between treated and untreated groups. *P*-values are indicated within each graph. Abbreviation: R.E., relative expression.

Our inability to discriminate between anti-tumor effects of the various treatments may reflect limitations of our analysis of tumor cell proliferation. While we have previously shown that the only proliferating (Ki67^+^) cells in the mouse *Nf1*-OPG are tumor cells,^20^ it is possible that we are not directly assessing tumor cell content. To address this possibility, we leveraged single-cell RNA sequencing to identify *Nf1*-OPG tumor-specific genes. By comparing differentially expressed gene (DEG) profiles of tumor cells to those of the other cell types in the tumors, we discovered *Neu4* and *Gpr17* as tumor-specific markers (Figure 4A). *Neu4* and *Gpr17* were expressed in >45% of the cells in the tumor cell cluster, <2% of the cells in the non-tumor cell clusters, with an average log_2_ fold change >4.5 (Figure 4B; Supplementary Figure 4A). Moreover, *NEU4* and *GPR17* expression was enriched in the tumor cell clusters of both sporadic and NF1-associated low-grade gliomas (pilocytic astrocytoma) (Figure 4C, D; Supplementary Figure 4B, C). Using independently generated mouse optic nerve samples, *Neu4* and *Gpr17* RNA expression was increased in *Nf1*^OPG^ mice relative to those from wild-type and *Nf1^+/-^* mice (Figure 4E). However, all treatments that reduced *Nf1*-OPG proliferation also exhibited decreased *Neu4* and *Gpr17* expression (Figure 4F), suggesting similar effects of the various treatments on tumor cell content.

### All Treatments Decrease TME Soluble Mediator Expression in *Nf1* ^OPG^ Mice

**Figure 5.**
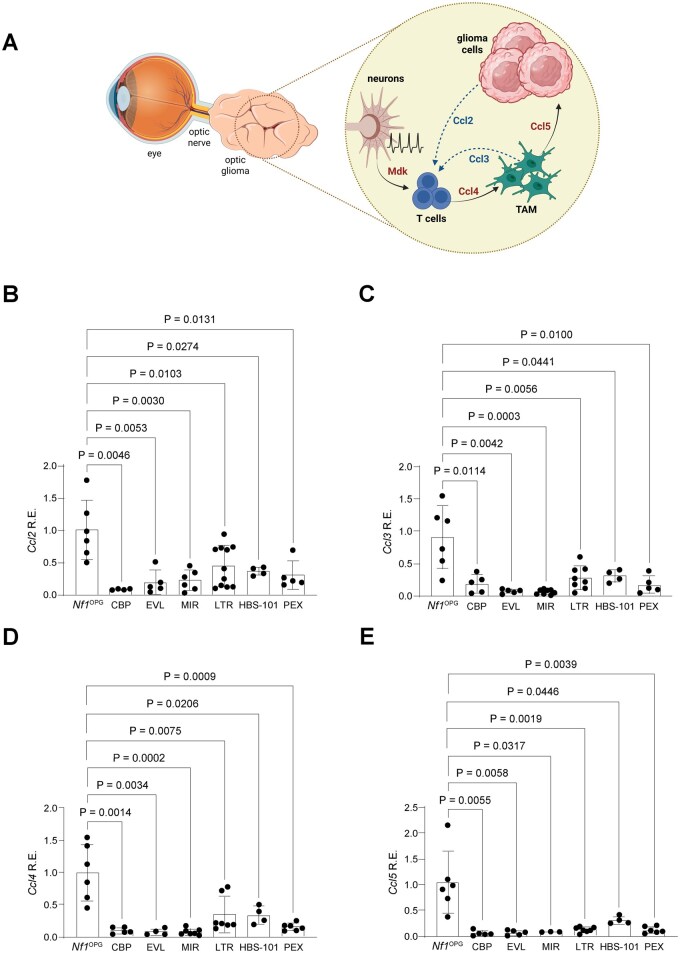
(A) Proposed model of Ccl2, Ccl3, Ccl4, and Ccl5 in the optic glioma TME circuit of *Nf1*-OPG mice. (B) *Ccl2* mRNA expression by qPCR in treated and untreated *Nf1*-OPG mice (*Nf1*^OPG^, *n* = 6 mice; CBP, *n* = 4 mice; EVL, *n* = 5 mice; MIR, *n* = 6 mice; LTR, *n* = 11 mice; HBS-101, *n* = 4 mice; PEX, *n* = 5 mice). (C) *Ccl3* mRNA expression by qPCR in treated and untreated *Nf1*-OPG mice (*Nf1*^OPG^, *n* = 6 mice; CBP, *n* = 5 mice; EVL, *n* = 5 mice; MIR, *n* = 8 mice; LTR, *n* = 8 mice; HBS-101, *n* = 4 mice; PEX, *n* = 5 mice). (D) *Ccl4* mRNA expression by qPCR in treated and untreated *Nf1*-OPG mice (*Nf1*^OPG^, *n* = 6 mice; CBP, *n* = 5 mice; EVL, *n* = 4 mice; MIR, *n* = 7 mice; LTR, *n* = 7 mice; HBS-101, *n* = 4 mice; PEX, *n* = 6 mice). (E) *Ccl5* mRNA expression by qPCR in treated and untreated *Nf1*-OPG mice (*Nf1*^OPG^, *n* = 6 mice; CBP, *n* = 5 mice; EVL, *n* = 5 mice; MIR, *n* = 3 mice; LTR, *n* = 7 mice; HBS-101, *n* = 4 mice; PEX, *n* = 6 mice). Data are represented as the mean ± SEM. (B-E), Two-tailed student’s *t*-test between treated and untreated groups. *P*-values are indicated within the graph. Abbreviation: R.E., relative expression. Figure illustrations (5A) were created using BioRender (BioRender.com)

We next hypothesized that our inability to prioritize treatments might reflect the differential effects of these therapies on the supportive tumor microenvironment (TME). Previous studies from our laboratory revealed striking *Nf1*-OPG tumor dependence on non-neoplastic tumor cells and soluble mediators (Figure 5A). In this manner, *Nf1*-mutant neurons initiate a paracrine stromal cascade in which midkine induces CD8^+^ T cells to produce Ccl4, which in turn stimulates TAM expression of Ccl5, a cytokine that increases glioma growth.^18^^,^^19^^,^^22^ Additionally, CD8^+^ T cells are recruited by either glioma cell-derived Ccl2 or TAM-produced Ccl3^26^,^27^

For this analysis, we quantified *Ccl2, Ccl3, Ccl4* and *Ccl5* mRNA levels by qPCR in *Nf1*^OPG^ mouse optic nerves following treatment with carboplatin (CBP), everolimus (EVL), mirdametinib (MIR), lamotrigine (LTR), HBS-101, and pexidartinib (PEX), compared to untreated controls. All the treatments reduced *Ccl2, Ccl3, Ccl4,* and *Ccl5* mRNA levels compared to untreated mice (Figure 5B-E). This finding is consistent with the notion that these low-grade gliomas function as integrated circuits, such that targeting different cells in the circuit results in similar effects on ecosystem paracrine factor production.

### Mirdametinib and Pexidartinib Did Not Restore RNFL Thickness

While neuro-oncologists focus on tumor size measurements (magnetic resonance imaging; MRI) to judge the efficacy of tumor treatments, there is an increasing emphasis being placed on functional outcomes.^28^ This is especially important in children with NF1-OPG, where visual decline is the most common indication for treatment.^10^ While vision in children is typically assessed using age-appropriate tests,^8^ a number of children with co-morbid attention deficits are unable to focus adequately for accurate assessments of vision and will require optical coherence tomography (OCT) to measure RNFL thickness as a surrogate for optic nerve fiber health.^8^

**Figure 6.**
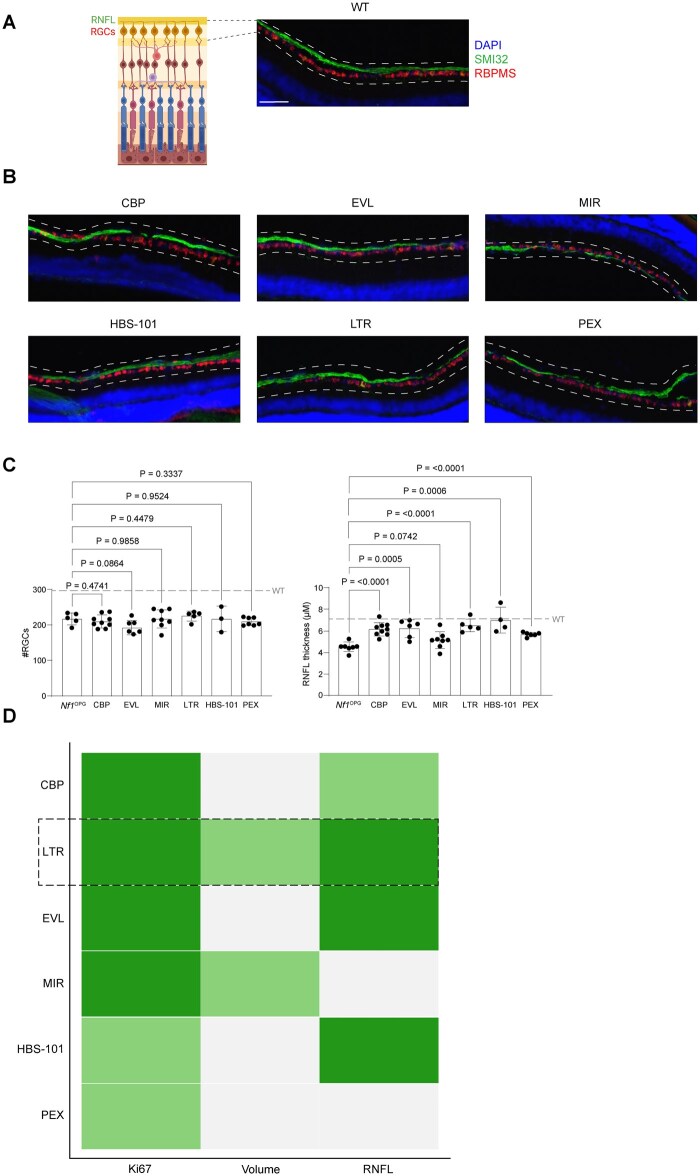
(A) Schematic representing the retinal nerve fiber layer (RNFL) and retinal ganglion cell (RGC) layer relative to immunofluorescence labeling of the RNFL (Smi32) and RGCs in a representative wild-type (WT) mouse eye. (B) Retinal ganglion cell (RGC; only female *Nf1*^OPG^ mice) count (number of RBPMS^+^ cells) from treated mice relative to untreated mice (*Nf1*^OPG^, *n* = 5 mice; CBP, *n* = 9 mice; EVL, *n* = 7 mice; MIR, *n* = 8 mice; LTR, *n* = 5 mice; HBS-101, *n* = 3 mice; PEX, *n* = 7 mice). Retinal nerve fiber layer (female *Nf1*^OPG^ mice) thickness (Smi-32^+^ fibers) from treated mice relative to untreated mice (*Nf1*^OPG^, *n* = 7 mice; CBP, *n* = 9 mice; EVL, *n* = 6 mice; MIR, *n* = 8 mice; LTR, *n* = 5 mice; HBS-101, *n* = 4 mice; PEX, *n* = 6 mice). Dotted lines denote average value of nerves from wild-type female *Nf1*^OPG^ mice. (D) A heatmap representing the fold change of Ki67^+^ cells, optic nerve volume, and RNFL thickness from treated relative to untreated *Nf1*^OPG^ mice. The green boxes in the heat map indicate the relative change in each of the parameters listed. Grey boxes denote a lack of statistically significant differences. Data are represented as the mean ± SEM. Scale bars, (B) 100 µm. (C), Two-tailed student’s *t*-test between treated groups and wild type. *P*-values are indicated within each graph. Figure illustrations (6A) were created using BioRender (BioRender.com).

For this reason, we assessed retinal nerve fiber layer (RNFL) thickness and retinal ganglion cell (RGC) content, which yield identical results in *Nf1*-OPG mice.^29^ In this analysis (Figure 6A), we limited our analysis to females, since *Nf1*-OPG-induced visual impairment is sexually dimorphic in mice.^30^^,^^31^ While RGC numbers were unchanged, RNFL thickness, as measured by the width of SMI32^+^ axonal fibers, was increased following carboplatin, everolimus, lamotrigine, and HBS-101, but not mirdametinib and pexidartinib, treatment, to wild-type levels (Figure 6C; Supplementary Figure 5A). It should be appreciated that RNFL thickness and RGC number are similar in wild-type and *Nf1*^+/-^ mice regardless of sex. The observed dissociation between RNFL thickness and RGC number reflects the progression of optic glioma-induced injury, both temporally and spatially, where axonal damage and RNFL thinning occur prior to RGC death.^29^

### Head-to-Head Preclinical Treatment Analysis Identifies Lamotrigine as a Lead Compound for Clinical Translation

Using head-to-head comparisons relative to the standard-of-care therapy (carboplatin) and no treatment, we found that everolimus, lamotrigine, and HBS-101 exhibited both anti-tumoral and neuroprotective effects (Figure 6D). In contrast, mirdametinib decreased tumor proliferation and optic nerve volume, without any effect on RNFL thickness, while pexidartinib only reduced optic glioma proliferation. However, only lamotrigine treatment resulted in reduced *Nf1*-OPG optic nerve proliferation, volume, and RNFL thinning. This observation suggests that lamotrigine might have the greatest potential efficacy as both an anti-tumoral and neuroprotective therapy for NF1-OPG in humans.

## Discussion

With the availability of robust preclinical models of human brain tumors, identifying promising therapies with a high likelihood of success in clinical trials remains a major challenge for the field. This is particularly important for low-grade gliomas,^3^ like NF1-OPG, where reduced survival is not a factor,^32^ time-honored treatments typically result in excellent tumor control,^33^ and preservation of neurologic and functional outcomes is key.^28^^,^^34^^,^^35^ For these reasons, we designed preclinical trials using human-relevant outcomes that aim to both attenuate tumor growth and improve retinal health. Leveraging this strategy, our findings raise several points relevant to other low-grade nervous system neoplasms.

First, NF1-OPG biology is dictated by the cooperative interactions of numerous non-cancerous cell types in the tumor microenvironment (TME). Prior studies from our laboratory have revealed at least two separable OPG-supportive stromal circuits involving the coordinated interplay of (1) neurons and oligodendrocyte precursors^36^ or (2) neurons, T cells, and TAMs.^18^^,^^19^^,^^22^ The first circuit requires light-induced activation of retinal ganglion cell (RGC) neurons and the release of ADAM10, which cleaves membrane-bound NLGN3 from OPCs to drive tumor initiation. In proof-of-concept preclinical experiments, blocking ADAM10 cleavage suppresses tumor growth (both volume and proliferation^36^) This finding is consistent with other preclinical studies in which a related inhibitor, INCB7839, reduced tumor growth malignant glioma growth and mouse mortality.^37^ Since the mechanisms underlying RGC activation and ADAM10 production are currently under active investigation and remain unknown, we chose to focus on the second better characterized neuronal circuit. Consistent with the principles of integrated circuit function, all treatments, regardless of their primary target cell, resulted in reduced paracrine mediator expression. As such, interruption of these interdependent nodes resulted in inhibition of paracrine factors that attract T cells (Ccl2, Ccl3) and those important for TAM support of tumor growth (Ccl4, Ccl5). Using single cell RNA sequencing, there was no evidence for the emergence of new cellular or paracrine factor adaptations (data not shown), supporting the idea that this low-grade glial neoplasm in mice can be equivalently targeted at multiple nodes within an established stromal circuit.

Second, we observed a dissociation between anti-tumoral and neuroprotective effects. This was previously underscored by the finding that *Nf1*-OPG mice of both sexes harbor tumors of equivalent size and proliferation rate, but only females exhibit reduced RGC content, RNFL thickness, and visual acuity.^30^ The female skewing of vision loss in the setting of OPG has also been reported in children with NF1.^30^ While the etiology for this sex imbalance in humans is unknown, the preferential vision loss in female *Nf1*-OPG mice results from estrogen receptor-mediated release of the neurotoxic paracrine factor, IL-1β, from glial cells.^31^^,^^38^ It should be noted that the differences in RNFL neuroprotection observed with the various treatments do not operate at the level of IL-1β production (Supplementary Figure 5B) and likely reflect other mechanisms that remain to be elucidated.

Third, the use of a preclinical design more reflective of the clinical setting with head-to-head comparisons permits a prioritization of candidates based on both anti-tumoral and neuroprotective outcomes. For these experiments, we specifically selected a treatment interval (6 to 12 weeks of age) during which the greatest increases in tumor proliferation and volume are observed, with little change thereafter.^20^^,^^29^ In addition, we used both standard neuropathological (%Ki67^+^ cells) and clinically relevant (optic nerve volume) measurements to approximate tumor growth. Optic nerve volumes were chosen based on prior studies demonstrating excellent concordance between gross histology and MRI.^39^ Similarly, SMI32 measurements of RNFL thickness were used as a biomarker to reflect objective estimates of visual loss (OCT), which were previously shown to be equivalent (*R*^2^=0.889).^29^ We chose not to incorporate behavior-based visual acuity assessments (eg virtual optokinetic system; VOS^30^), as these are complicated by attention deficits in *Nf1*-mutant mice,^40^ similar to children with NF1.

Using this head-to-head referential preclinical design, comparisons were made to heterozygous *Nf1*-mutant (*Nf1+/-*) and wild-type (WT) mice, as well as to time-honored standard of care carboplatin-treated^6^^,^^41^ and untreated *Nf1*-OPG mice. This is important, since heterozygous *Nf1*-mutant mice exhibit increased optic nerve volumes,^39^ similar to some children with NF1,^42^ and harbor increased numbers of monocytes, unrelated to tumor formation, relative to WT mice.^43^ It should be noted that none of the treatments restored optic nerves to WT levels, arguing that treatments during the period of optic glioma evolution do not prevent tumor formation (glioma initiation).^36^ Instead, we observed varying effects of the various treatments on optic nerve proliferation, volume, and tissue architectural distortion. In this regard, carboplatin treatment resulted in reduced tumor proliferation, decreased tissue architectural distortion, and near-WT RNFL thickness, similar to that seen with everolimus, HBS-101, and pexidartinib treatment. While HBS-101 and pexidartinib treatments have not been evaluated in human NF1-OPG clinical trials, prior studies with everolimus revealed stable or improved radiological responses in 11/18 children and stable or improved visual acuity in 12/14 children.^35^ HBS-101, a novel midkine inhibitor, reduced optic nerve proliferation, normalized RNFL thickness, and decreased the expression of tumor (*Neu4*, *Gpr17*) and TME (*Ccl2*, *Ccl3*, *Ccl4*, *Ccl5*) genes, supporting its continued development as a targeted therapy for pediatric NF1-associated glioma. In contrast, mirdametinib was effective only as an anti-neoplastic agent with no improvement in RNFL thinning in *Nf1*-OPG mice. Children experiencing vision loss from optic pathway gliomas frequently demonstrate a decline in RNFL thickness.^44^ Although RNFL thickness is often used as a surrogate marker of vision, the exact relationship between VA and RNLT thickness has not been completely elucidated. Early clinical studies evaluating the use of selumetinib, an alternative MEK inhibitor, in children with both NF1 and non-NF1-associated recurrent/relapsed OPG revealed vision stabilization or partial improvement,^45^ however, RNFL thickness was not assessed. While both anti-neoplastic and neuroprotective effects were previously reported by our laboratory using a much higher dose of mirdametinib (5mg/kg twice daily^5^), raising the possibility that dose escalation may afford some degree of neuroprotection, this could be difficult given the dose limiting toxicities observed in many MEKi phase 1 trials.^46^

Importantly, only lamotrigine exhibited both anti-neoplastic and neuroprotective properties with doses routinely used in clinical practice for managing epilepsy in children,^47^ as previously reported in mice,^18^ suggesting that lamotrigine warrants further consideration as a promising treatment for children with NF1-OPG. Repurposing anti-epileptic and mood stabilizing drugs for neuronal targeting in the setting of cancer forms the basis for cancer neuroscience therapeutics.^48^ As such, previous preclinical studies have revealed key roles for cholinergic,^49^ glutaminergic,^50^ and GABA-ergic^51^ neuronal control of malignant brain tumor growth. In this regard, the proposed use of lamotrigine for NF1-OPG therapy underscores the value of interfering with neuron-cancer interdependence.

While this study is limited by a lack of long-term follow-up and multiple rounds of treatment, the proposed experimental design framework offers unique opportunities to directly compare therapies for their abilities to halt tumor progression and visual decline. It should be noted that prior preclinical studies revealed durable effects of a single round of lamotrigine treatment with respect to tumor growth and RNFL thinning more than 3 months following the cessation of therapy.^18^ Moreover, setting a higher bar for clinical translation might result in more successful results in the clinical workplace for pediatric neuro-oncological diseases characterized by good overall survival, a low tolerance for significant adverse effects, and a pressing need for therapies that attenuate secondary neuronal dysfunction and improve tumor-associated morbidity.

## Supplementary Material

vdaf215_Supplementary_Data

## Data Availability

All RNA sequencing data have been deposited in GEO (GSE303054) and are publicly available.
