# Supplementary material for: Head-to-head preclinical treatment design prioritizes promising therapies for neurofibromatosis type 1 optic glioma clinical translation
Source: Neurooncol Adv. 2025 Oct 4;7(1):vdaf215. doi: 10.1093/noajnl/vdaf215 (PMC12768503; doi:10.1093/noajnl/vdaf215)
Supplement: vdaf215_Supplementary_Data [file vdaf215_supplementary_data.zip › NOA-D-25-00333R1_Supplementary Materials- Clean.docx]

**Head-to-head preclinical treatment design prioritizes promising therapies for Neurofibromatosis 1 optic glioma clinical translation**

Talia Eligator^1^, Jit Chatterjee^1^, Shintaro Yamada^1^, Anthony Kirchner ^1^, Hareesh B. Nair^2^, Jason R. Fangusaro^3^, David H. Gutmann^1^

^1^Department of Neurology, Washington University, St. Louis MO 63110; ^2^Department of Molecular and Translational Medicine, Texas Tech University Health Science Center, Rick Francis St, Texas 79905; ^3^Department of Pediatrics and Winship Cancer Center, Emory University School of Medicine, Atlanta GA 30322

**Supplementary Figures**

Supplementary Figure S1

Supplementary Figure S2

Supplementary Figure S3

Supplementary Figure S4

Supplementary Figure S5

**Supplementary Tables**

Supplementary Table 1

Supplementary Table 2

Supplementary Table 3


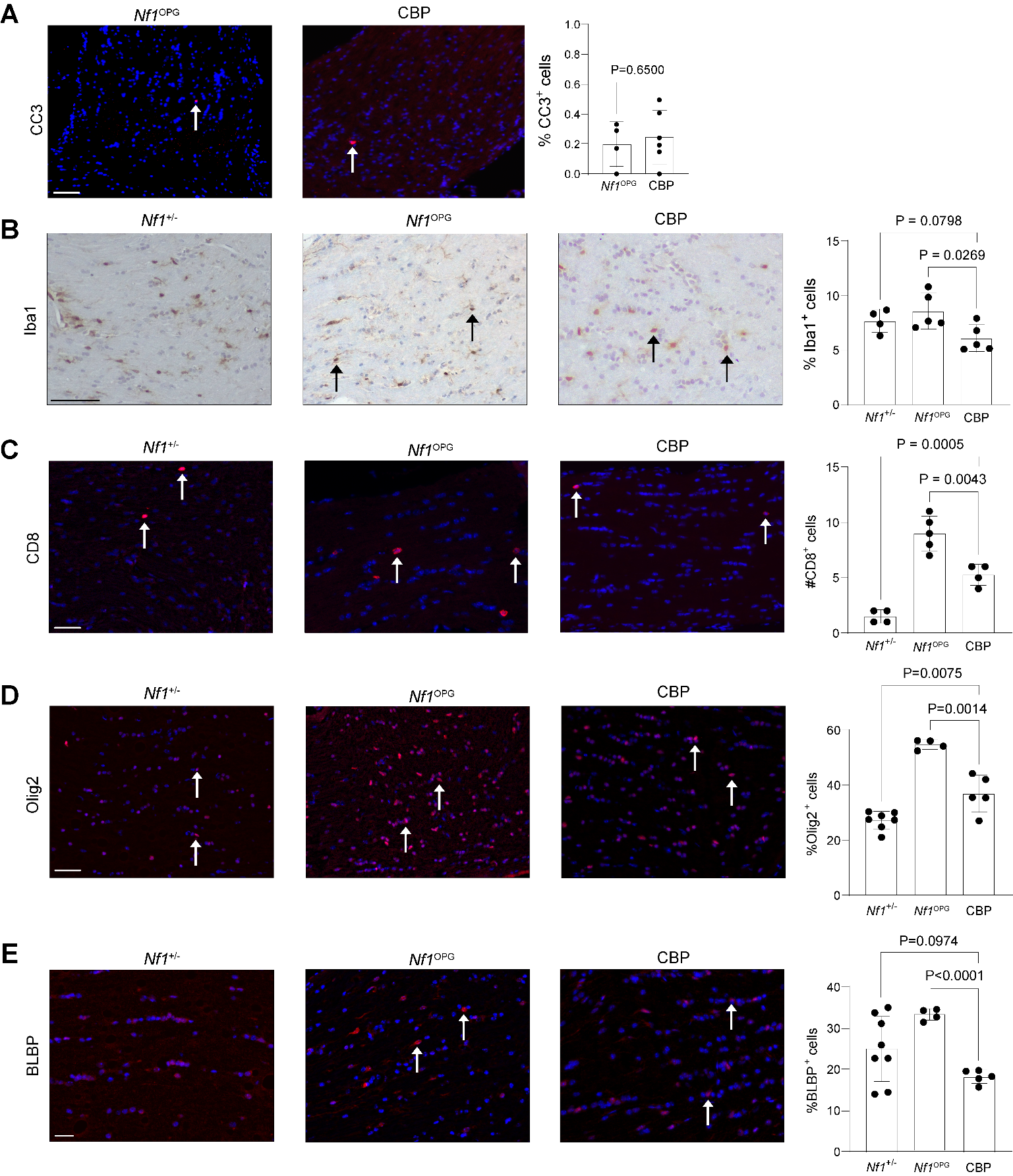


**Supplementary Figure 1**

*Nf1*^OPG^ mice were treated from six to twelve weeks of age with carboplatin (Sigma-Aldrich C2538; 15mg/kg IP injection once weekly). Optic nerves were analyzed after euthanasia at 12 weeks of age. Immunohistochemistry (IHC) or immunofluorescence (IF) and quantification of (**A**) cleaved caspase 3^+^ (percent cleaved caspase-3^+^ cells; %CC3^+^ cells; *Nf1*^OPG^, n=4 mice; CBP, n=5 mice). (**B**) tumor-associated monocytes (TAM; %Iba1^+^ cells in brown; *Nf1*^+/-^, n=4 mice; *Nf1*^OPG^, n=5 mice; CBP, n=5 mice). (**C**) CD8^+^ T cells (number of CD8^+^ cells in red; *Nf1*^+/-^, n=4 mice; *Nf1*^OPG^, n=5 mice; CBP, n=4 mice). (**D**) Oligodendrocyte lineage cells (%Olig2^+^ cells in red; *Nf1*^+/-^, n=4 mice; *Nf1*^OPG^, n=4 mice; CBP, n=5 mice). (**E**) Blbp^+^ cell content (%Blbp^+^ cells in red; *Nf1*^+/-^, n=3 mice; *Nf1*^OPG^, n=4 mice; CBP, n=5 mice). Scale bars, **A-E**, 40µm. Data are represented as the mean ± SEM. **A-E**, Two-tailed student’s *t*-test between treated and untreated groups. *P* values are indicated within each graph. **Abbreviations**: CBP, carboplatin; IP, intraperitoneal; IHC, immunohistochemistry; IF, immunofluorescence; *Nf1*^OPG^, no treatment.


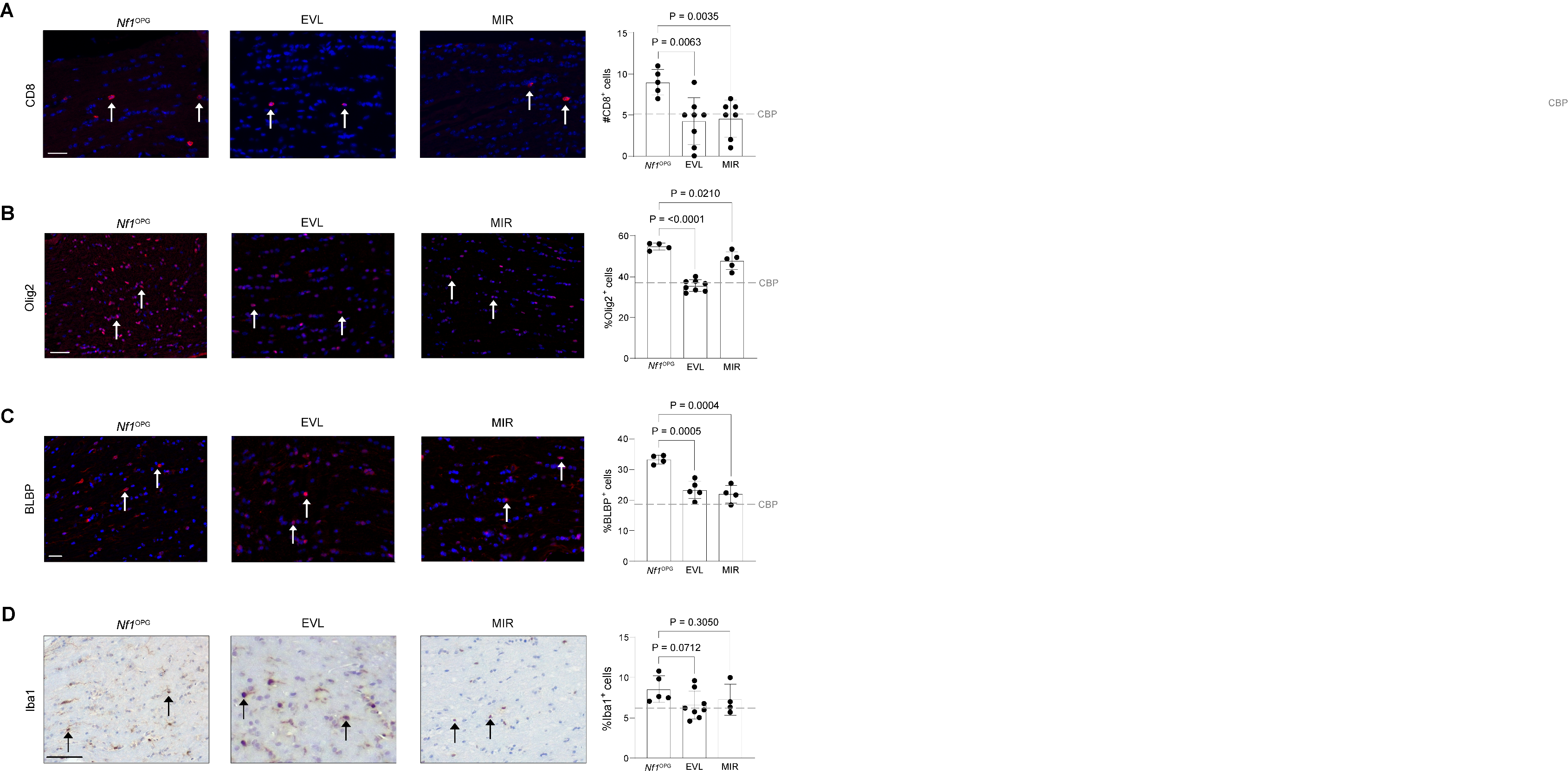


**Supplementary Figure 2**

*Nf1*^OPG^ mice were treated from six to twelve weeks of age with everolimus (Selleck Chemicals S1120; 1.7 mg/kg gavage for 5days each week) or mirdametinib (STEMCELL Technologies 72184; 2.5mg/kg oral gavage twice daily for five days each week). Optic nerves were analyzed after euthanasia at 12 weeks of age. Immunohistochemistry (IHC) or immunofluorescence (IF) and quantification of (**A**) CD8^+^ T cells (number of CD8^+^ cells in red; *Nf1*^OPG^, n=5 mice; EVL, n=8 mice; MIR; n=7 mice). (**B**) Oligodendrocyte lineage cells (%Olig2^+^ cells in red; *Nf1*^OPG^, n=4 mice; EVL, n=8 mice; MIR, n=5 mice). (**C**) Blbp^+^ cell content (%Blbp^+^ cells in red; *Nf1*^OPG^, n=4 mice; EVL, n=5 mice; MIR, n=4 mice). (**D**) tumor-associated monocytes (TAM; %Iba1^+^ cells in brown; *Nf1*^OPG^, n=5 mice; EVL, n=8 mice; MIR, n=4 mice). Dotted lines denote the average values from carboplatin (CBP)-treated mice. Scale bars, **A-D**, 40µm. Data are represented as the mean ± SEM. **A-D**, Two-tailed student’s *t*-test between treated and untreated groups. *P* values are indicated within each graph. **Abbreviations**: CBP, carboplatin; EVL, everolimus; MIR, mirdametinib; IHC, immunohistochemistry; IF, immunofluorescence; *Nf1*^OPG^, no treatment


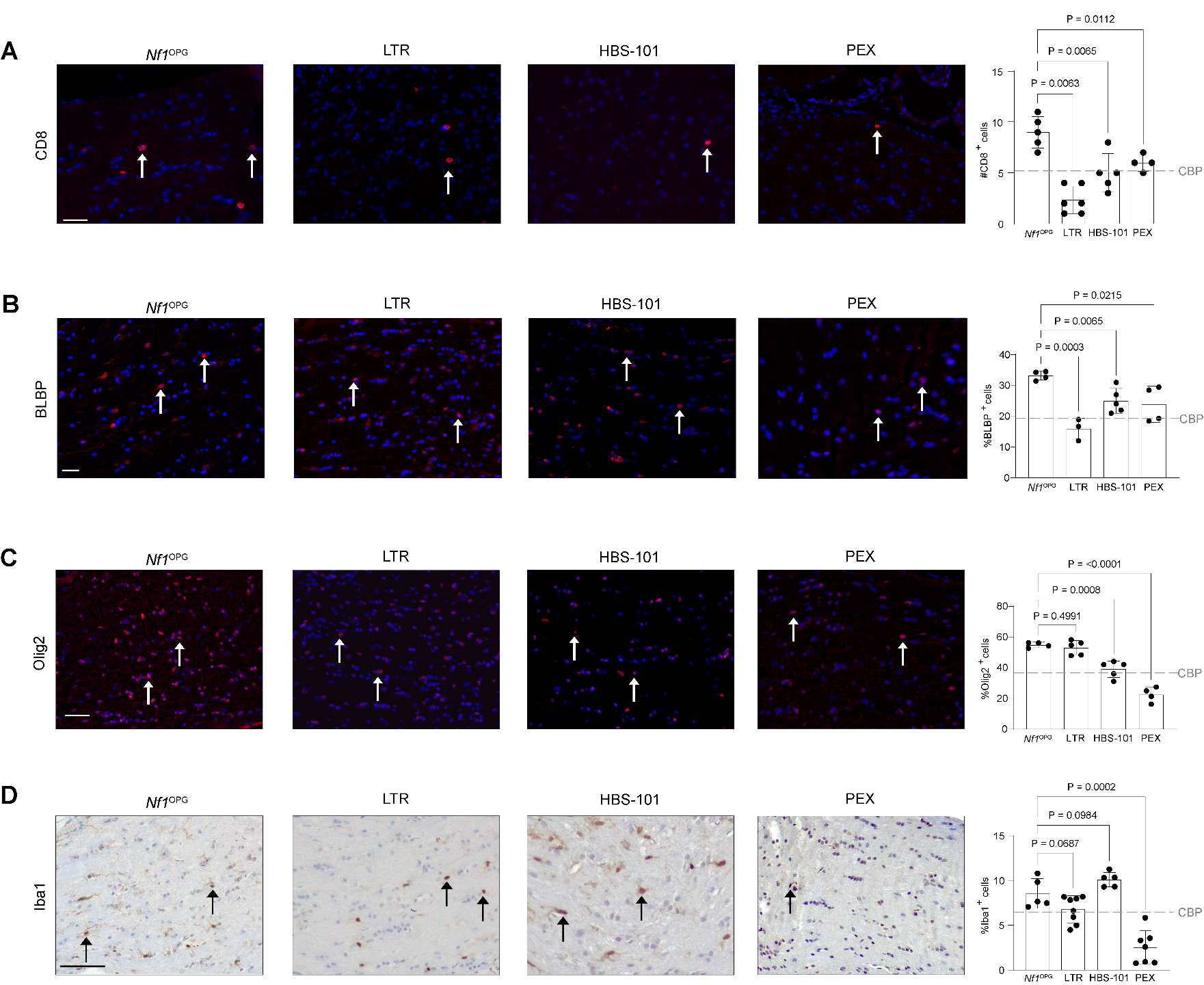


**Supplementary Figure 3**

*Nf1*^OPG^ mice were treated from six to twelve weeks of age with lamotrigine (Selleck Chemicals S3024; 2.5mg/kg gavage twice daily for five days each week), HBS-101 (20mg/kg IP for 5 days each week) or pexidartinib (Medchem HY-16749A; 275 mg/kg PLX3397-containing chow daily). Optic nerves were analyzed after euthanasia at 12 weeks of age. Immunohistochemistry (IHC) or immunofluorescence (IF) and quantification of (**A**) CD8^+^ T cells (number of CD8^+^ cells in red; *Nf1*^OPG^, n=5 mice; LTR, n=6 mice; HBS-101, n=5 mice; PEX, n=4 mice). (**B**) Blbp^+^ cell content (%Blbp^+^ cells in red; *Nf1*^OPG^, n=4 mice; LTR, n=4 mice; HBS-101, n=5 mice; PEX, n=4 mice). **(C)** Oligodendrocyte lineage cells (%Olig2^+^ cells in red; *Nf1*^OPG^, n=4 mice; LTR, n=5 mice; HBS-101, n=5 mice; PEX, n=4 mice). (**D**) tumor-associated monocytes (TAM; %Iba1^+^ cells in brown; *Nf1*^OPG^, n=5 mice; LTR, n=8 mice; HBS-101, n=5 mice; PEX, n=7 mice). Dotted lines denote the average value from carboplatin (CBP)-treated mice. Scale bars, **A-D**, 40µm. Data are represented as the mean ± SEM. **A-D**, Two-tailed student’s *t*-test between treated and untreated groups. *P* values are indicated within each graph. **Abbreviations**: CBP, carboplatin; IP, intraperitoneal; LTR, lamotrigine; *Nf1*^OPG^, no treatment; PEX, pexidartinib; IHC, immunohistochemistry; IF, immunofluorescence.


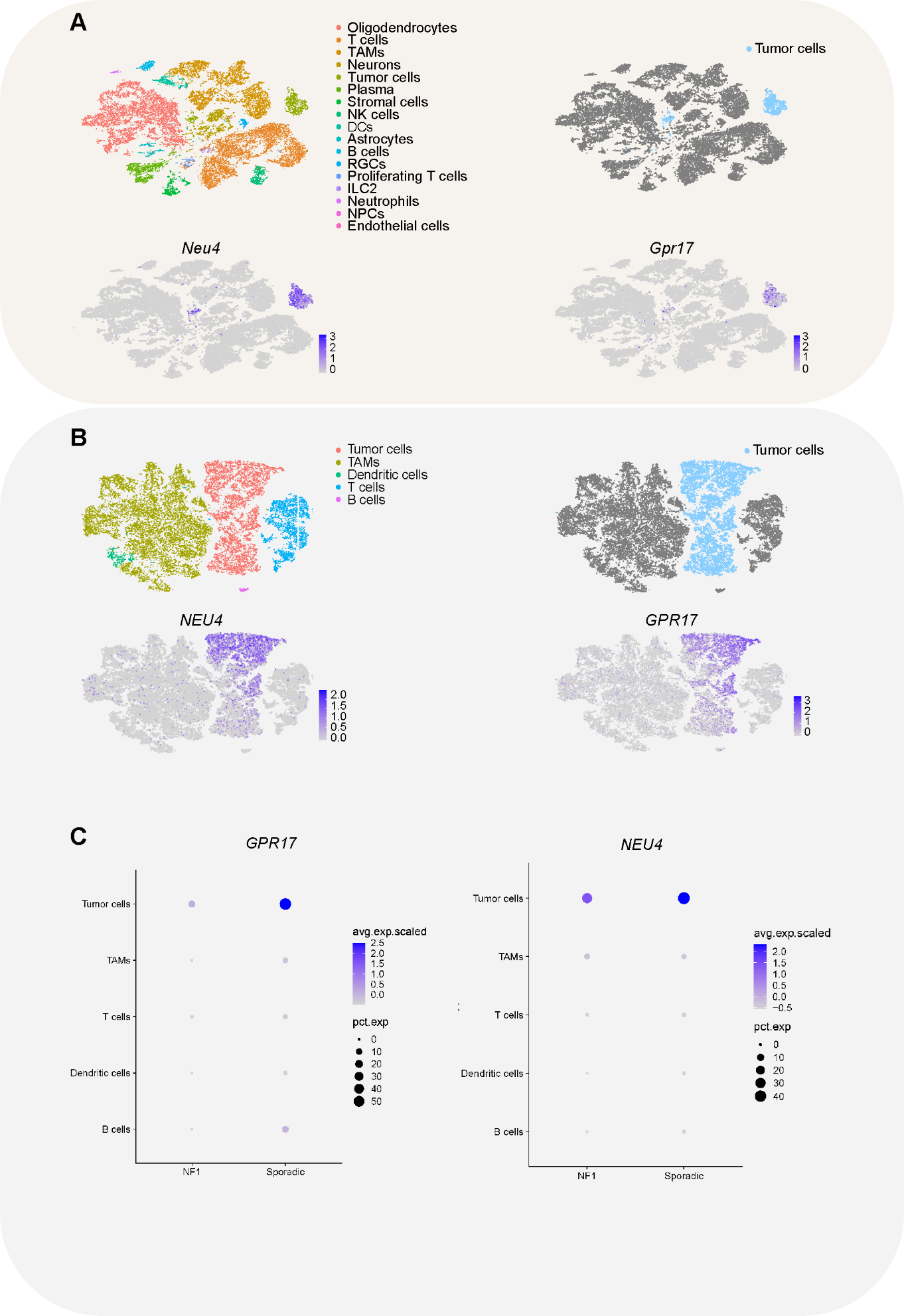


**Supplementary Figure 4**

t-SNE visualization plot showing single-cell RNA sequencing results from (**A**) *Nf1*^OPG^ mice and (**B**) human PA tumors highlighting *Neu4* and *Gpr17* expression. (**C**) Dot plot showing *NEU4* and *GPR17* expression in sporadic (n=4) and NF1-associated (n=1) human PA tumors.


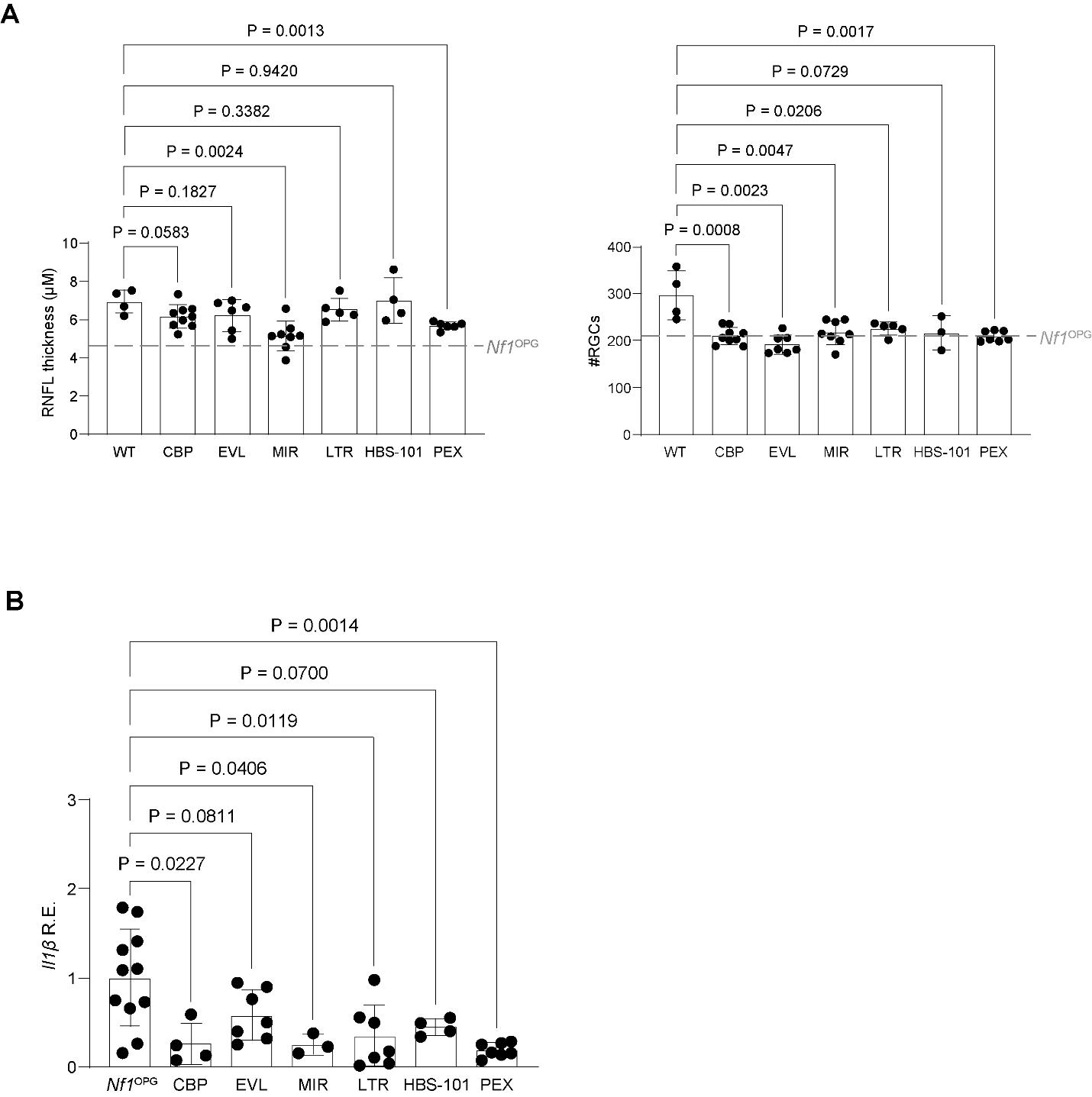


**Supplementary Figure 5**

(**A**) Immunofluorescence (IF) of treated female *Nf1*-OPG mouse retinae. Dotted lines in the photomicrographs outline the RGCs and RNFL in female *Nf1*^OPG^ mice. (**C**) Retinal nerve fiber layer (RNFL; only female *Nf1*^OPG^ mice) thickness (Smi32^+^ fibers in green) from treated mice relative to wild-type mice (WT, n=4 mice; CBP, n=9 mice; EVL, n=6 mice; MIR, n=8 mice; LTR, n=5; HBS-101, n=4 mice; PEX, n=6 mice). Retinal ganglion cell (RGC in red; only female *Nf1*^OPG^ mice) count (number of RBPMS^+^ cells) from treated mice relative to wild-type mice (WT, n=4 mice; CBP, n=9 mice; EVL, n=7 mice; MIR, n=8 mice; LTR, n=5 mice; HBS-101, n=3 mice; PEX, n=7 mice). Dotted lines denote the average values from untreated (*Nf1*^OPG^) female *Nf1*^OPG^ mice. (**B**) *Il1b* mRNA expression across treated groups (*Nf1*^OPG^, n=11 mice; CBP, n=4 mice; EVL, n=7 mice; MIR, n=3 mice; LTR, n=7 mice; HBS-101, n=4 mice; PEX, n=7 mice). It should be appreciated that RNFL thickness and RGC number in the retinae of WT and *Nf1^+/-^* mice are indistinguishable. Data are represented as the mean ± SEM. **A**-**B,** Two-tailed student’s *t*-test between treated and untreated groups. *P* values are indicated within the graph. R.E., relative expression. **Abbreviations**: CBP, carboplatin; EVL, everolimus; LTR, lamotrigine; MIR, mirdametinib; *Nf1*^OPG^, no treatment; PEX, pexidartinib
