# Supplementary material for: Head-to-head preclinical treatment design prioritizes promising therapies for neurofibromatosis type 1 optic glioma clinical translation
Source: Neurooncol Adv. 2025 Oct 4;7(1):vdaf215. doi: 10.1093/noajnl/vdaf215 (PMC12768503; doi:10.1093/noajnl/vdaf215)
Supplement: vdaf215_Supplementary_Data [file vdaf215_supplementary_data.zip › NOA-D-25-00333R1_Supplementary Table 2.docx]

**Supplementary Table 2. Mouse qRT-PCR primers used**

| **Gene** | **Probe set** |
| --- | --- |
| *Ccl2* | Mm00441242_m1 (TaqMan Gene Expression) |
| *Ccl3* | Mm00441259_g1 (TaqMan Gene Expression) |
| *Ccl4* | Mm00443111_m1 (TaqMan Gene Expression) |
| *Ccl5* | Mm01302427_m1 (TaqMan Gene Expression) |
| *Gapdh* | Mm99999915_g1 (TaqMan Gene Expression); internal control |
| *Gpr17* | Mm02619401_s1 (TaqMan Gene Expression) |
| *Neu4* | Mm00620597_m1 (TaqMan Gene Expression) |
