# Supplementary material for: Head-to-head preclinical treatment design prioritizes promising therapies for neurofibromatosis type 1 optic glioma clinical translation
Source: Neurooncol Adv. 2025 Oct 4;7(1):vdaf215. doi: 10.1093/noajnl/vdaf215 (PMC12768503; doi:10.1093/noajnl/vdaf215)
Supplement: vdaf215_Supplementary_Data [file vdaf215_supplementary_data.zip › NOA-D-25-00333R2_Supplementary Table 1.docx]

| **Antibody** | **Host** | **Source** | **Dilution** |
| --- | --- | --- | --- |
| Anti-mouse Alexa Fluor 488 (IF) | goat | Invitrogen, A11029 | 1:200 |
| Anti-rabbit Alexa Fluor 568 (IF) | goat | Invitrogen, A11011 | 1:200 |
| Biotinylated anti-mouse (IHC) | goat | Vector Laboratories, BA-9200 | 1:200 |
| Biotinylated anti-rabbit (IHC) | goat | Vector Laboratories, BA-1000 | 1:200 |
| BLBP (IF) | rabbit | Millipore Sigma, ABN-14 | 1:400 |
| CD8 (IF) | rabbit | Cell Signaling, D4W2Z | 1:500 |
| Cleaved Caspase-3 (IF) | rabbit | Cell Signaling- 9661S | 1:200 |
| Iba1 (IHC) | rabbit | Wako, 019-19741 | 1:500 |
| Ki67 (IHC) | mouse | BD Biosciences, ab15580 | 1:400 |
| Olig2 (IF) | rabbit | GeneTex, GTX132732 | 1:500 |
| RBPMS (IF) | rabbit | ProteinTech, 15187-1-AP | 1:400 |
| Smi32 (IF) | mouse | BioLegend, 801701 | 1:300 |

**Supplementary Table 1. Antibodies Used**

**Abbreviations**: IF, immunofluorescence, IHC, immunohistochemistry
